# Vancomycin Monitoring for Treatment of Acute Pulmonary Exacerbations of Adult Cystic Fibrosis Patients

**DOI:** 10.1155/pm/5683225

**Published:** 2025-05-28

**Authors:** Darrell Smith, James Sanders, Marguerite Monogue

**Affiliations:** ^1^Department of Pharmacy, University of Texas Southwestern Medical Center, Dallas, Texas, USA; ^2^Division of Infectious Diseases and Geographic Medicine, University of Texas Southwestern Medical Center, Dallas, Texas, USA

**Keywords:** acute kidney injury, acute pulmonary exacerbation, area under the curve, cystic fibrosis, vancomycin

## Abstract

**Background:** Therapeutic drug monitoring (TDM) for vancomycin (VAN) in adult people with cystic fibrosis (pwCF) historically has utilized trough concentrations. Recent VAN TDM guidelines recommend area under the curve (AUC) monitoring to reduce the risk of acute kidney injury (AKI), despite limited evidence to support this practice in adult pwCF.

**Methods:** This single-center, retrospective, observational cohort study included 143 adult pwCF admitted from July 1, 2017, to July 1, 2022, with an acute pulmonary exacerbation that received VAN for at least 72 h with available VAN plasma concentrations for TDM for AUC (*n* = 39) or trough monitoring (*n* = 104). Eligible patients with multiple hospital admissions during the study period were incorporated as separate encounters. The primary outcome was the incidence of AKI.

**Results:** Receipt of concurrent nephrotoxins was more common in the AUC cohort than in the trough cohort (97% vs. 81%, *p* = 0.01), but the rate of AKI was similar (7.7% vs. 10.6%, *p* = 0.76). AUC monitoring was associated with earlier achievement of TDM goal (median 0 days (IQR 0–2) vs. 2 days (IQR 0–4), *p* < 0.01), lower total daily doses (34.8 mg/kg/day (IQR 27.6–49) vs. 57.5 mg/kg/day (IQR 43.9–68.6), *p* < 0.01), and fewer regimen changes (median 1 change (IQR 0–2) vs. 2 changes (IQR 1–3), *p* < 0.01). In patients with MRSA, pulmonary function recovery, readmission, and mortality were similar.

**Conclusion:** In adult pwCF, the incidence of AKI was similar between AUC and trough monitoring cohorts; however, AUC monitoring achieved therapeutic targets sooner with fewer regimen modifications without significantly increasing the number of concentrations compared to trough monitoring.

**Summary**

• In adult pwCF patients, the incidence of AKI was similar between AUC and trough monitoring cohorts; however, AUC monitoring achieved therapeutic targets sooner with fewer regimen modifications without significantly increasing the number of concentrations compared to trough monitoring.

## 1. Introduction

Cystic fibrosis (CF) is a life-shortening, autosomal recessive disease. Mutations in the genes encoding for the cystic fibrosis transmembrane conductance regulator (CFTR) protein cause dysfunctional transport of chloride ions. This affects several body systems, but morbidity and mortality are driven mainly by progressive lung disease involving mucous retention, chronic colonization, and inflammation [1, 2]. Methicillin-resistant *Staphylococcus aureus* (MRSA) is a common organism associated with CF-related lung colonization and infections; albeit the percentage of people with CF (pwCF) colonized with MRSA has decreased from 26% in 2016 to 18% in 2021 [3]. There is a higher tendency for pwCF to become *S. aureus* carriers possibly due to a defense defect against the bacteria and/or the bacteria's ability to grow well in anaerobic conditions, such the hypoxic environment of a pwCF's mucus plugged respiratory tract [4]. MRSA in lung cultures has been associated with decreases in forced respiratory volume in 1 s (FEV1) and increased risk of mortality [5–7].

The presence of anti-MRSA therapy, such as vancomycin, is often part of the antimicrobial arsenal used for the treatment of CF acute pulmonary exacerbations (APEs). Antimicrobial dosing is optimized through the utilization of pharmacokinetic (PK) and pharmacodynamic (PD) indices, which describe antimicrobial efficacy in relation to exposure. Physiologic changes in pwCF have led to reported differences in the PK of antibiotics compared to people without CF [8]. The effect of CF on PK varies among classes of antibiotics, but generally, pwCF have higher volumes of distribution and increased drug clearance, which may necessitate higher loading and maintenance doses [8]. However, a small PK analysis of vancomycin in 10 adults with CF found that the disposition and PK of vancomycin were similar in adults with CF and healthy volunteers [9].

The PK/PD index that is best associated with vancomycin efficacy and toxicity is the ratio of area under the curve (AUC) to the minimum inhibitory concentration (MIC). Historically, vancomycin troughs have been used to estimate AUC exposures, yet data show that troughs are an imprecise surrogate for AUC values in both pwCF and those without [10, 11]. Vancomycin troughs of 15–20 mg/L usually achieve a daily AUC of at least 400 mg∗h/L; however, there is variability in the upper extent of daily vancomycin exposure, which may lead to nephrotoxicity [12]. Studies suggest that the risk of AKI increases when troughs are maintained above 15 mg/L [13] or if daily AUC exposures exceed 650–1300 mg∗h/L [14–17]. AUC-guided dosing has been shown to reduce the incidence of nephrotoxicity compared with trough monitoring in patients without CF [18]. For this reason, guidelines now recommend that vancomycin doses be based on AUC exposures in patients with serious MRSA infections, with a target AUC/MIC exposure of 400–600 mg∗h/L to balance efficacy and toxicity [12]. These guidelines have limited recommendations on vancomycin dosing for pwCF [12]. Similarly, the 2009 Cystic Fibrosis Pulmonary Guidelines Treatment of Pulmonary Exacerbations do not address the use of vancomycin in this population [19]. There are limited data comparing trough versus AUC vancomycin monitoring in pwCF in regard to the latter's safety benefit [20, 21]. Therefore, the aim of this study was to determine the difference in clinical outcomes between trough and AUC monitoring of vancomycin in the adult pwCF population that our institution serves.

## 2. Materials and Methods

### 2.1. Study Design

This single-center, retrospective, observational cohort study included adult pwCF admitted to the University of Texas Southwestern (UTSW) Medical Center in Dallas, Texas, from July 1, 2017, to July 1, 2022, with an APE that received vancomycin for at least 72 h with available vancomycin plasma concentrations for therapeutic drug monitoring (TDM). Patients were excluded if they had a history of lung transplantation, required renal replacement therapy at vancomycin initiation, or had acute kidney injury (AKI) at vancomycin initiation. Eligible patients with multiple hospital admissions during the study period were incorporated as separate encounters. Patients with a past medical history of CF, APE, and intravenous (IV) vancomycin receipt were identified with ICD-10-CM codes and eRx ID codes. This study was approved by the UTSW Institutional Review Board.

Patients were then subdivided into either the AUC or trough cohort, depending on which monitoring method was used during the admission. The majority of AUC encounters were after March 2020, which was a few months after the vancomycin consensus guideline update. Prior to this, our institution targeted troughs of 15–20 mg/L in the adult pwCF population. Vancomycin AUC was calculated at the time of vancomycin therapy via trapezoidal methodology by utilizing two vancomycin serum concentrations in a single dosing interval with a goal AUC of 400–600 mg∗h/L. Study data was collected and managed using REDCap (Research Electronic Data Capture) electronic data capture tools hosted at UTSW [22, 23].

The primary outcome was the incidence of AKI, which was defined as an increase in serum creatinine of ≥ 0.5 mg/dL or an increase in SCr 1.5× the baseline value on two consecutive measurements within 48-h period since the last vancomycin exposure [12]. Secondary outcomes included inpatient vancomycin duration, total vancomycin duration, hospital length of stay, percent achievement of the TDM goal defined as a trough of 15–20 mg/L or an AUC of 400–600 mg∗h/L, incidence of supratherapeutic exposure to vancomycin defined as an exposure above the TDM goal, incidence of vancomycin accumulation defined as a supratherapeutic exposure after being in the therapeutic range previously without a regimen change, number of vancomycin concentrations for TDM, number of changes to the dose or frequency of the vancomycin regimen, total daily dose to achieve the TDM goal, and time to a regimen that achieved the TDM goal.

A subgroup efficacy analysis was performed in patients who met the following criteria: MRSA in the sputum 3 months prior to or 3 weeks after hospitalization; did not receive alternative anti-MRSA therapy (i.e., linezolid, ceftaroline, doxycycline, or trimethoprim/sulfamethoxazole) for 48 h at any point during the admission; available baseline, admission, and discharge FEV1% predicted. Definitions of FEV1% predicted were as follows: baseline FEV1% predicted was the maximum FEV1% predicted recorded in the electronic medical record within 6 months of the index hospitalization; admission FEV1% predicted was the FEV1% predicted ±72 h of the index hospitalization; discharge FEV1% predicted was the highest FEV1% predicted between 72 h prior to hospital discharge and 30 days after hospital discharge. Subgroup efficacy outcomes included length of stay, 30-day readmission, 30-day mortality, return to 90% of baseline FEV1% predicted, absolute change in FEV1% predicted, and relative change in FEV1% predicted defined as absolute change in FEV1% predicted divided by baseline FEV1% predicted.

### 2.2. Statistical Analysis

Statistical analysis was performed via GraphPad Prism Version 9.5.1 for Windows (GraphPad Software Inc., San Diego, California, United States) with descriptive statistics, Fisher's exact test for categorical variables, and Mann–Whitney *U* test for continuous variables. A *p* value of < 0.05 was considered statistically significant.

## 3. Results

**Figure 1:**
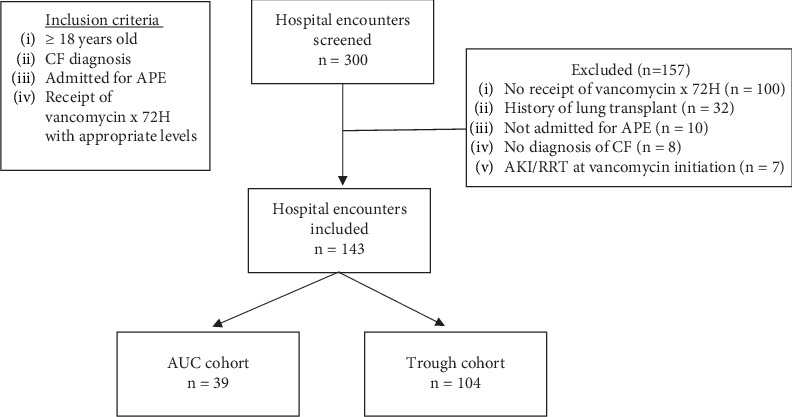
Patient selection for study inclusion. Abbreviations: APE = acute pulmonary exacerbation; CF = cystic fibrosis; AKI = acute kidney injury; RRT = renal replacement therapy.

A total of 300 patient encounters were screened, and 143 encounters were included in the analysis. The most common reason for exclusion was not receiving vancomycin for 72 h (Figure 1). Of the 143 patients, 39 patients' vancomycin therapy was monitored via AUC and 104 patients were monitored via trough concentrations.  Table 1 shows the baseline characteristics of the two groups. Patients in the AUC cohort were older, weighed more, had a higher BMI, and had a higher incidence of concurrent nephrotoxins compared to the trough cohort. IV aminoglycosides (49.0%), piperacillin/tazobactam (27.3%), IV contrast dye (25.2%), and nonsteroidal anti-inflammatory drugs (23.1%) were the most common nephrotoxins among the patients included in the study (*n* = 143). Baseline serum creatinine was 0.7 mg/dL in both cohorts, and a similar number of patients in AUC cohort (23.1%) and trough cohort (27.9%) had estimated creatine clearance > 130 mL/minute at admission using the Cockcroft–Gault equation. A similar number of patients in each cohort were homozygous for the Phe508del CFTR gene (48.7% and 54.8% of patients in the AUC and trough cohort, respectively). CFTR modulators were more commonly used in the AUC cohort (97.4%) than in the trough cohort (80.8%) (*p* = 0.04). All patients were treated with additional antibiotics and APE medications that may affect FEV1% predicted.

Of the 143 patients included in the study, 3 (7.7%) in the AUC cohort and 11 (10.6%) in the trough cohort met the primary outcome of AKI (*p* = 0.76) (Table 2). Secondary outcomes are also shown in Table 2. There was no difference in vancomycin duration and length of stay between groups. Additionally, the percent of achieving goal TDM, incidence of supratherapeutic vancomycin exposure, incidence of accumulation of vancomycin, and the number of vancomycin concentrations for TDM were similar between groups. The median number of regimen changes (i.e., a change to dose and/or frequency) during admission was lower in the AUC cohort compared to the trough cohort (median 1 change (IQR 0–2) vs. 2 changes (IQR 1–3), respectively, *p* < 0.01). The median (IQR) total daily dose to achieve TDM goal was lower in the AUC cohort (34.8 mg/kg/day (IQR 27.6–49) vs. 57.5 mg/kg/day (IQR 43.9–68.6), *p* < 0.01), and the time to a regimen that achieved TDM goal was shorter in the AUC cohort compared to the trough cohort (median 0 days (0–2) vs. 2 days (0–4), *p* < 0.01). These results were previously published as an abstract at ID Week 2023 [24].

**Figure 2:**
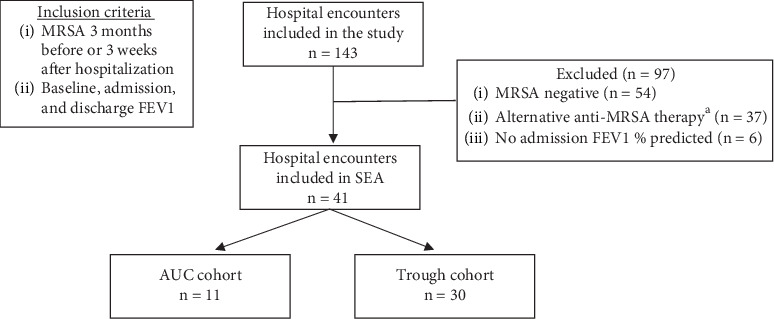
Patient selection for subgroup efficacy analysis. ^a^Ceftaroline, linezolid, doxycycline, or trimethoprim/sulfamethoxazole for 48 h. Abbreviations: MRSA = methicillin-resistant *S. aureus*; SEA = subgroup efficacy analysis.

Of the 41 patients included in the subgroup efficacy analysis, 11 patients (26.8%) were monitored via AUC and 30 patients (73.2%) were monitored via troughs (Figure 2). There were no differences in length of stay, 30-day readmission, 30-day mortality, return to baseline FEV1% predicted, absolute change in FEV1% predicted, or relative change in FEV1% predicted (Table 3).

## 4. Discussion

In adult pwCF, the rates of AKI were similar between vancomycin TDM methods, but vancomycin AUC monitoring resulted in a therapeutic regimen sooner at lower total daily vancomycin doses and fewer number of regimen changes without significantly increasing the number of vancomycin concentrations for TDM compared to trough monitoring. Previous studies support AUC monitoring over trough monitoring for its safety benefit (i.e., incidence of AKI [18]), but data in adult CF patients are limited [20]. The efficacy benefit of AUC monitoring compared with trough monitoring is less clear clinically given trough-based monitoring tends to overshoot the intended AUC target, which is the PK/PD index best associated with efficacy. In this study, rates of AKI were numerically lower in the AUC cohort despite the AUC cohort having a higher incidence of concurrent nephrotoxin use (7.7% vs. 10.6%, respectively, *p* = 0.76). This finding is consistent with a similar study from Mitchell et al., who found comparable rates of AKI when adult pwCF were monitored via AUC (*n* = 40) and trough (*n* = 73) (12% vs. 16%, respectively, *p* = 0.58) [20]. However, they found that less severely graded AKIs, as defined by KDIGO Guidelines [25], occurred with AUC monitoring since the only two incidences of Grade 2–3 AKIs were observed in the trough cohort. The overall higher incidences of AKI observed in that study compared to ours may be explained by the lower threshold in SCr rise used by Mitchell et al. to define AKI, especially since all but two AKIs were Grade 1. A similar study performed by Oermann et al. in pediatric pwCF also did not observe a difference in nephrotoxicity between monitoring methods although specific definitions or rates are not stated [21].

In this study, patients monitored with AUC had a shorter time to a therapeutic regimen and often had an empiric regimen that was therapeutic (median 0 days, IQR 0–2), which is consistent with the findings of Oermann et al. [21]. They found the mean time to a therapeutic concentration to be significantly shorter with AUC monitoring (mean 28.4 ± 25.98 h) compared to trough monitoring (mean 86.3 ± 75.8 h) (*p* < 0.01, 95%CI = 21.2–100.5) [21]. A potential confounding factor to these findings is the practice of vancomycin dosing for pwCF at our institution. During an initial patient admission, standardized weight-based vancomycin dosing is used and adjusted based on TDM. For subsequent admissions, this information would be used to guide the vancomycin dosing strategy with the goal of achieving a therapeutic regimen sooner.

One theoretical risk of AUC dosing would be the increase in the number of phlebotomies for TDM, but this was not seen in this study nor the study by Oermann et al. (mean 4, SD ± 2 in both trough and AUC cohorts) [21]. This could be due to the fewer number of vancomycin regimen changes seen in the AUC cohort since a regimen change would warrant additional vancomycin concentrations to be measured. Similarly, Oermann et al. found a higher percent of TDM goal attainment with AUC monitoring (95%) versus trough monitoring (43%) (*p* < 0.01) which presumably led to fewer dosing regimen changes and subsequent phlebotomies [21].

There does not appear to be a difference in clinical efficacy between the two monitoring methods in our limited subgroup efficacy analysis. A study by Fusco et al. in pediatric CF patients failed to identify a correlation between vancomycin trough concentrations or AUC/MIC exposures and change in pulmonary function tests results or return to baseline FEV1% predicted [11]. In contrast, Mitchell et al. found an overall significant difference in return to baseline FEV1% predicted with AUC monitoring (80%) compared to trough monitoring (61%) (*p* = 0.02); however, this is confounded by higher rates of CFTR modulator use in the AUC cohort, particularly in adults with CF [20]. Additionally, this benefit was not observed in the pediatric subgroup where CFTR modulator use was similar [20].

Limitations of this study include the retrospective design and small sample size. A prespecified power calculation was not performed, and this study may not have been adequately powered to detect a true difference in outcomes. This is also true for the subgroup efficacy analysis. AKIs were not stratified based on severity, which may have provided additional insight into the degree of kidney injury. There were high rates of concurrent nephrotoxins in each cohort that confounds vancomycin as the sole agent responsible for an AKI. Notably, there were significant differences in baseline characteristics between groups, as well as group imbalance in regard to the number of patients in each cohort. Specific trough values were not collected for either cohort, which limits the ability to make a between-group comparison of vancomycin exposure. Lastly, data points to classify severity of infection and urinary creatinine were not collected, so it is unclear whether the phenomenon of augmented renal clearance (ARC) was present in this study.

In conclusion, the incidence of AKI in adult pwCF was similar between AUC and trough monitoring cohorts; however, AUC monitoring achieved therapeutic targets sooner with fewer regimen modifications without significantly increasing the number of concentrations compared to trough monitoring. In a limited cohort, there was not a difference in clinical outcomes. Additional data are needed to solidify the benefits of AUC- over trough-based vancomycin dosing.

## Figures and Tables

**Table 1:** Baseline characteristics (*n* = 143).

**Characteristic**	**AUC cohort (** **n** = 39**)**	**Trough cohort (** **n** = 104**)**	**p** ** value**⁣^∗^
Male [*n*, %]	26 (66.7)	56 (53.9)	0.19
MRSA positive^a^ [*n*, %]	21 (53.9)	68 (65.4)	0.25
Age (years) [median (IQR)]	33 (28–35)	25 (21–31)	< 0.01
Weight (kg) [median(IQR)]	64.4 (57–72.6)	57.7 (48.8–66.2)	< 0.01
BMI (kg/m^2^) [median (IQR)]	22.2 (20.4–26.8)	20.2 (17.8–24.2)	< 0.01
Baseline serum creatinine (mg/L) [median (IQR)]	0.7 (0.6–0.8)	0.7 (0.6–0.8)	0.29
Baseline FEV1% predicted (%) [median (IQR)]	55 (32–79)	55 (37–72)	0.85
Delta F508 homozygous genotype [*n*, %]	19 (48.7)	57 (54.8)	0.57
CFTR modulator^b^ [*n*, %]	26 (66.7)	49 (47.1)	0.04
Concurrent nephrotoxins^c^ [*n*, %]	38 (97.4)	84 (80.8)	0.01
Other antibiotics^d^ [*n*, %]	39 (100)	104 (100)	1
APE medications^e^ [*n*, %]	39 (100)	104 (100)	1

Abbreviations: APE, acute pulmonary exacerbation; BMI, body mass index; CFTR, cystic fibrosis transmembrane conductance regulator; FEV1, forced expiratory volume in 1 s; MRSA, methicillin-resistant *S. aureus*.

^a^MRSA positive from sputum or bronchoscopy cultures.

^b^CFTR modulator: ivacaftor, lumacaftor/ivacaftor, tezacaftor/ivacaftor, elexacaftor/tezacaftor/ivacaftor.

^c^Nephrotoxins: IV aminoglycoside, piperacillin/tazobactam, IV contrast dye, amphotericin, nonsteroidal anti-inflammatory drugs, colistin, loop diuretics, thiazide diuretics, angiotensin II receptor antagonist, angiotensin-converting enzyme inhibitor, and phenylephrine.

^d^Other antibiotics: beta lactams, fluoroquinolones, macrolides, colistin, aminoglycosides, trimethoprim/sulfamethoxazole, tetracyclines, and linezolid.

^e^APE medications: albuterol, hypertonic saline, dornase alfa, ibuprofen, inhaled aminoglycoside, inhaled colistin, and corticosteroids.

⁣^∗^Fisher's exact test for categorical data and Mann–Whitney *U* test for continuous variables.

**Table 2:** Primary and secondary outcomes.

**Variable**	**AUC cohort (** **n** = 39**)**	**Trough cohort (** **n** = 104**)**	**p** ** value**⁣^∗^
Primary outcome			
Acute kidney injury^a^ [*n*, %]	3 (7.7)	11 (10.6)	0.76
Secondary outcomes			
Inpatient vancomycin duration [median (IQR)]	10 (5–14)	8.5 (5–12)	0.10
Total vancomycin duration [median (IQR)]	11 (8–14)	12 (8.3–14)	0.81
Length of stay [median (IQR)]	12 (8–14)	10.5 (7–14)	0.24
Goal TDM achieved^b^ [*n*, %]	31 (79.5)	80 (76.9)	0.82
Supratherapeutic exposure^c^ [*n*, %]	20 (51.3)	58 (55.8)	0.71
Accumulation of vancomycin^d^ [*n*, %]	9 (23.1)	32 (30.8)	0.41
Number of vancomycin concentrations [median (IQR)]	4 (3–6)	4 (3–7)	0.80
Number of regimen changes [median (IQR)]	1 (0–2)	2 (1–3)	< 0.01
TDD to achieve TDM goal (mg/kg/day) [median (IQR)]	34.8 (27.6–49)	57.5 (43.9–68.6)	< 0.01
Time to regimen that achieved TDM goal (days) [median (IQR)]	0 (0–2)	2 (0–4)	< 0.01

Abbreviations: IQR, interquartile range; SCr, serum creatinine; SD, standard deviation; TDD, total daily dose; TDM, therapeutic drug monitoring.

^a^An increase in SCr of ≥ 0.5 mg/dL or an increase in SCr 1.5× the baseline value on two consecutive measurements within 48-h period since the last vancomycin exposure.

^b^Vancomycin trough 15–20 mg/L or AUC 400–600 mg∗h/L.

^c^Vancomycin trough > 20 mg/L or AUC > 600 mg∗h/L.

^d^Vancomycin trough > 20 mg/L or AUC > 600 mg∗h/L after achieving goal trough/AUC level previously.

⁣^∗^Fisher's exact test for categorical data and Mann–Whitney *U* test for continuous variables.

**Table 3:** Subgroup efficacy analysis (*n* = 41).

**Variable**	**AUC cohort (** **n** = 11**)**	**Trough cohort (** **n** = 30**)**	**p** ** value**⁣^∗^
Length of stay (days) [median (IQR)]	14 (10–14)	10 (7–14)	0.22
30-day readmission [*n*, %]	2 (18.2)	8 (27.6)	0.69
30-day mortality [*n*, %]	0 (0)	1 (3.3)	1
Return to 90% of baseline FEV1% predicted [*n*, %]	10 (90.9)	24 (82.8)	0.66
Absolute change in FEV1% predicted (%) [median (IQR)]	7 (4–13)	9.4 (2.5–16)	0.74
Relative change in FEV1% predicted (%) [median (IQR)]	13.3 (10–21.5)	14.3 (8.3–21.9)	0.88

Abbreviations: FEV1, forced expiratory volume in 1 s; IQR, interquartile range; SD, standard deviation.

⁣^∗^Fisher's exact test for categorical data and Mann–Whitney *U* test for continuous variables.

## Data Availability

The chart review data used to support the findings of this study are available from the corresponding author upon request.

## Nomenclature

CF: cystic fibrosis
APE: acute pulmonary exacerbation
MRSA: methicillin-resistant *Staphylococcus aureus*
AKI: acute kidney injury
CFTR: cystic fibrosis transmembrane conductance regulator
% FEV1: percent predicted of forced expiratory volume at 1 s
UTSW: University of Texas Southwestern
EMR: electronic medical record
IQR: interquartile range
AUC: area under the curve
TDM: therapeutic drug monitoring
pwCF: person with cystic fibrosis
PK: pharmacokinetics
PD: pharmacodynamics
MIC: minimum inhibitory concentration
